# Live Imaging of Shoot Meristems on an Inverted Confocal Microscope Using an Objective Lens Inverter Attachment

**DOI:** 10.3389/fpls.2017.00773

**Published:** 2017-05-19

**Authors:** Zachary L. Nimchuk, Tony D. Perdue

**Affiliations:** ^1^Department of Biology, University of North Carolina at Chapel Hill, Chapel HillNC, USA; ^2^Curriculum in Genetics and Molecular Biology, University of North Carolina at Chapel Hill, Chapel HillNC, USA; ^3^Biology Microscope Core, University of North Carolina at Chapel Hill, Chapel HillNC, USA

**Keywords:** live imaging, meristem, laser scanning confocal microscopy, technology, plant development

## Abstract

Live imaging of above ground meristems can lead to new insights in plant development not possible from static imaging of fixed tissue. The use of an upright confocal microscope offers several technical and biological advantages for live imaging floral or shoot meristems. However, many departments and core facilities possess only inverted confocal microscopes and lack the funding for an additional upright confocal microscope. Here we show that imaging of living apical meristems can be performed on existing inverted confocal microscopes with the use of an affordable and detachable InverterScope accessory.

## Introduction

Above ground tissues in plants are derived from meristems, dynamic structures which harbor stem cell populations which self-renew but also spawn cells that differentiate to form the body of the plant (Soyars et al., 2016). The ability to live image growing plant meristems has provided unique insights into plant developmental processes. Real time imaging has allowed the dissection of dynamic processes such as cell lineage generation (Reddy et al., 2004; Reddy and Meyerowitz, 2005), gene regulatory networks (Heisler et al., 2005; Nimchuk et al., 2015), subcellular protein dynamics (Nimchuk et al., 2011; Yadav et al., 2011), mechanical properties (Hamant et al., 2008), and organogenesis (Besnard et al., 2014).

Live imaging growing shoot apical meristems (SAMs), or floral meristems (FMs), using confocal microscopy poses some unique technical challenges (Prunet et al., 2016). Plant shoots are negatively gravitropic and as such will rapidly re-orient upon inversion making live tracking of growing SAMs difficult. Inversion of SAMs also induces alterations in auxin transport, synthesis, and perception in the shoot (Morita and Tasaka, 2004). Auxin signaling is also involved in primordia formation in meristems (Galvan-Ampudia et al., 2016), potentially creating undesired impacts. Thus, continuous live imaging of intact SAMs and FMs is not possible on inverted confocal microscope configurations. SAMs and FMs can be detached and imaged for several hours. In some cases this might be sufficient to glean necessary data. However, for longer term imaging, detached meristems must be maintained viable, and often this is achieved through the use of media containing plant growth hormones (Fernandez et al., 2010; Besnard et al., 2014), raising the potential for artifacts. One other challenge with either detached or intact SAM imagining is the need to prevent damage and desiccation (Prunet et al., 2016). The SAM is fragile and even slight contact with coverslips will induce considerable tissue damage. Because primordia extend above the plane of the SAM, getting the SAM close enough to a coverslip to be in the focal range, without touching a coverslip, can be challenging. In addition, imaging and tissue maintenance requires an aqueous environment (Prunet et al., 2016). This prevents tissue desiccation, but also allows focal imaging and the ability to add experimental chemical treatments such as hormones, peptides, or chemical inducers. Inverted SAMs can be imaged using a drop of water acting as a water column between the SAM and a water immersion lens designed for use without a coverslip, or a “dipping” lens. However, the column conferred by the droplet is highly unstable and doesn’t fully protect against desiccation. As such, the current optimal arrangement for imaging SAM and FM tissue is to use an upright confocal configuration equipped with a water immersion “dipping” lens to image growing SAMs in plants temporarily submerged in an aqueous medium within a chamber (Prunet et al., 2016). In our experience getting a SAM in the proper focal plane without damaging the tissue on our Zeiss LSM710 is possible for only 10–20% of the SAMs dissected, a considerable failure rate that limits the ability to image effectively on our inverted confocal microscope.

Inverted confocal microscopes are the standard for many departments and core facilities owing to their broad preference in many animal, cell, and microbial imaging applications. As such, plant biologists often only have access to inverted confocal microscopes. Without the considerable personal or departmental funding support required to purchase an upright confocal microscope, options can be limited for plant biologists. Here we show that a standard inverted confocal microscope can be reliably and robustly used to image growing SAMs through the use of an objective lens inverter attachment arm. The InverterScope allows live imaging of SAMs in fully submerged aqueous environments, and is easily removable from the main microscope stand when not in use. All imaging was performed in this study using an inverted Zeiss LSM710 confocal microscope, but the InverterScope can be used on any inverted confocal series. No additional tissue processing or collection is specifically necessary for InverterScope use beyond what is already standard in the field (Prunet et al., 2016). In addition, the InverterScope is affordable and provides a cost effective solution for plant biologists to image growing above ground tissues. We recently published a paper using the InvertScope to analyze receptor signaling pathways in the SAM (Nimchuk, 2017), here we provide additional detail on the use and setup of the InverterScope for imaging SAM tissue.

## Results

### Configuring the Confocal Microscope System for Use with an Objective Lens Inverter

Before attaching the InverterScope all lasers should be turned off during the attachment procedure. The InverterScope alters the laser light path and the removal of lens while the laser is on is not advised in this procedure or any other.

**FIGURE 1:**
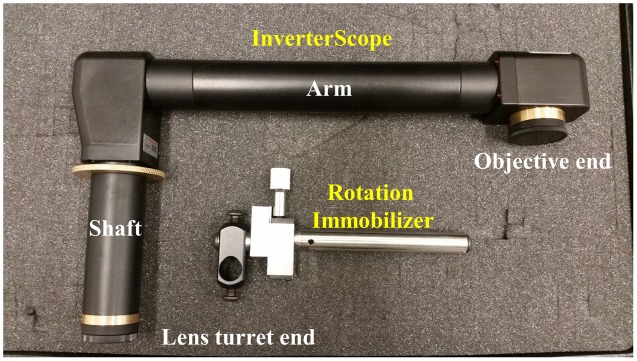
**The InverterScope and Rotation Immobilizer.** The InverterScope and Rotation Immobilizer are labeled in yellow, with the parts of the InverterScope discussed in the text labeled in white. The InverterScope comes in a protective cushioned lock case for storage when not in use (pictured). The jointed bend at the objective end of the InverterScope can rotate around the arm axis.

**FIGURE 2:**
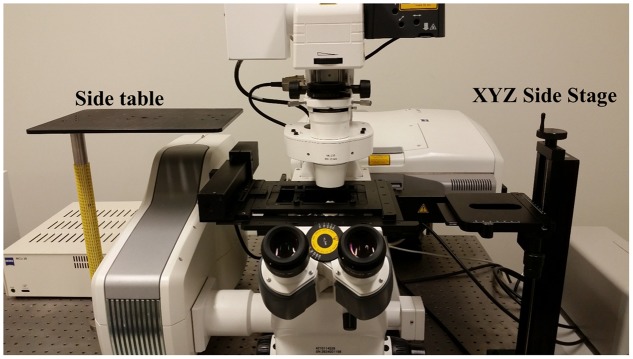
**Accessory table configuration on the microscope air table.** Use of the InverterScope requires two accessory side tables with our Zeiss LSM710 inverted microscope (pictured), which can be purchased from LSM Tech or constructed separately. Both are attached to the standard air table and need not be detached when the InverterScope is not in use. The side platform table is optional but allows the removed condenser to be displaced without fully disconnecting the condenser from the microscope (**Figure 3**).

The Zeiss LSM710 confocal microscope used in these studies is affixed to a standard Newport Scientific Grade optical breadboard air table with mounting holes to attach equipment. The InverterScope objective inverter was purchased from LSM Tech^1^. Several InverterScope configurations are available and we selected the 400S model, with a M27 thread size which is compatible with our objective lens turret (see below). A Rotation Immobilizer was also purchased with the InverterScope (**Figure 1**, see below). The 400S InverterScope configuration is a jointed tube which acts to bend the light path from the inverted configuration to an upright configuration through the use of internal reflective mirrors positioned in the two jointed bends. One end of the InverterScope is screwed into the opening on the lens turret while the objective lens is mounted on the receiver end (**Figure 1**), thus returning the objective lens to an upright configuration. As such, the arm of the InverterScope extends past the main microscope stage and necessitates the use of an adjustable XYZ Side Stage adjacent the microscope. For the configuration used at the UNC Biology Microscopy Core, a height adjustable XYZ Side Stage was purchased and was mounted onto the air table adjacent to a Zeiss AxioObserverZ.1 microscope (**Figure 2**). The XYZ Side Stage did not interfere with the use of the microscope when the InverterScope was not attached to the microscope stand (**Figure 2**). The exact positioning of the side stage could depend on the configuration of individual confocal microscopes. The side stage used in our configuration features a hand wheel based *Z*-axis height adjuster for coarse stage adjustment (see below). A second, optional side platform table was also purchased for use when mounting the InverterScope (see below).

### Attaching the InverterScope to the Confocal Microscope

Below we detail the steps taken to affix the InverterScope to the microscope. Completion of all steps takes about 2 min during either assembly or disassembly. At all steps, care is taken when handling microscope components. We have detached and re-attached the InverterScope on our Zeiss LSM710 several dozen times in a single year with no negative effects on confocal function in any imaging configuration.

### Step 1: Displacing the Microscope Condenser

**FIGURE 3:**
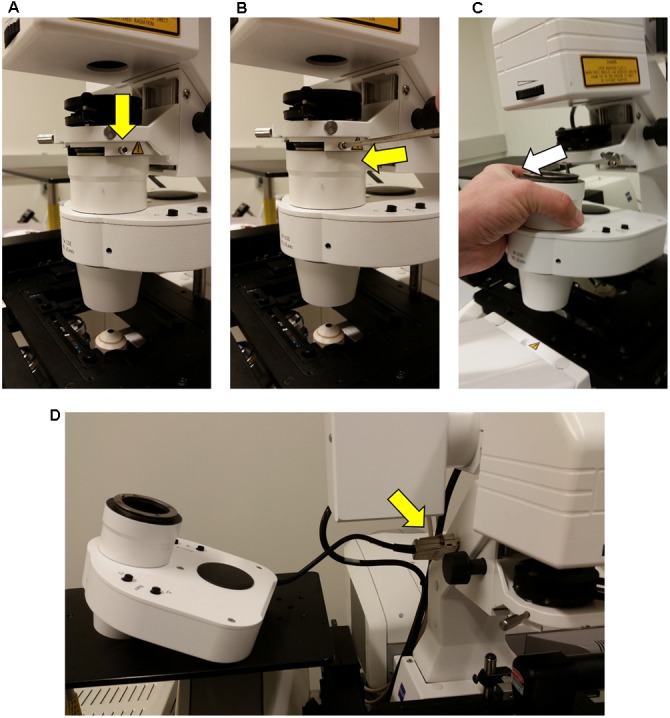
**Detachment of the condenser from the Zeiss LSM710 microscope.** In order to accommodate the InverterScope, the microscope condenser must be displaced from the Zeiss LSM710. This is accomplished by loosening, but not removing, the stabilizing screw [yellow arrow, **(A,B)**], and gently sliding the condenser unit out **(C)**. The condenser is then placed on side table **(D)**, without the need to detach the cable connections (yellow arrow).

When attached the InverterScope extends vertically from the lens turret above the plane of the main microscope stage (**Figure 7**). As such, this necessitates displacing the condenser above the main stage to accommodate the InverterScope (**Figure 3**). Zeiss Axio Observer series microscopes are modular in nature and the condenser can be removed easily by loosening the main mounting screws on the condenser mount and sliding the condenser out (**Figure 3**). There is no need to completely remove the mounting screw, loosening it to the point that the condenser can be gently slid out is sufficient and prevents the potential for losing the mounting screws. The condenser is also attached to the main microscope via communication cables but we have found that it is not necessary to fully detach the condenser. Instead the condenser is placed on a side platform table (**Figures 2**, **3C**), still attached to the microscope by the communication cables, but out of the way of the InverterScope. We have detached and reattached the condenser on our Zeiss LSM710 with no negative effects on confocal function in any imaging configuration.

### Step 2: Attaching the Rotation Immobilizer

**FIGURE 4:**
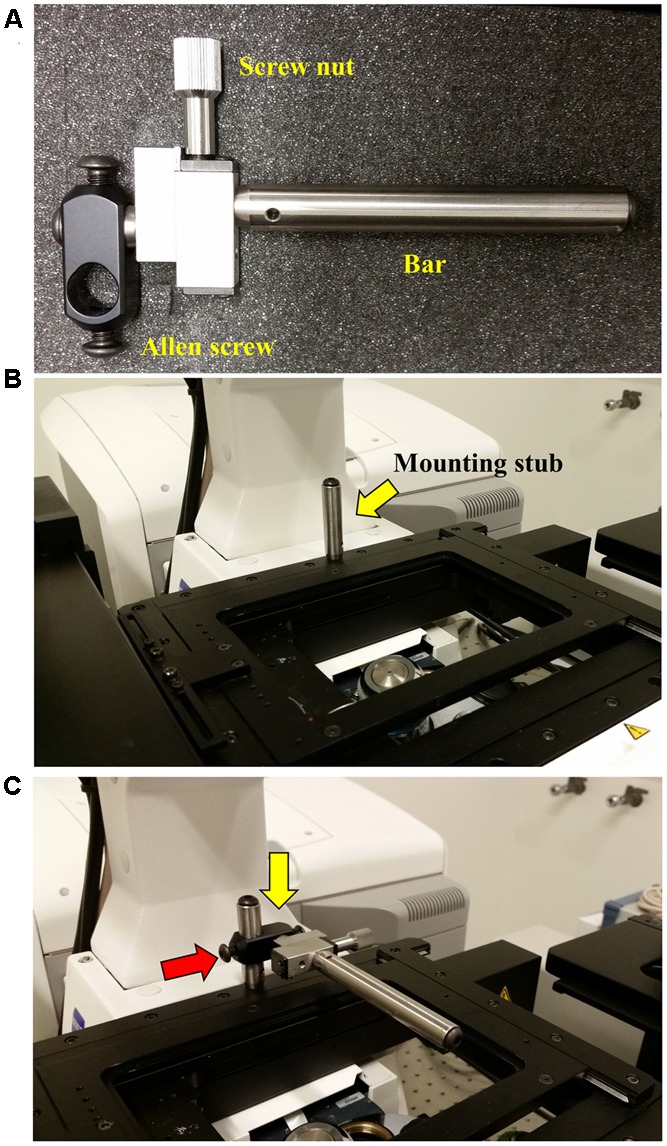
**Attaching the Rotation Immobilizer.** To prevent the attached InverterScope from drifting or torqueing the lens turret when attached a Rotation Immobilizer is used to stabilize the InverterScope. **(A)** The parts of the Rotation Immobilizer. **(B)** Position of the mounting stub on the microscope stage. The mounting stub is riveted into the microscope stage and comes with the Rotation Immobilizer. The mounting stub is permanent and does not interfere with microscope operation. **(C)** Attaching the Rotation Immobilizer to the mounting stub. The Rotation Immobilizer is slid on to the mounting stub (yellow arrow) and the Allen screw is tightened (red arrow) until the Rotation Immobilizer is stable on the mounting stub. Do not over tighten. The affixed Rotation Immobilizer should be about 0.5 inches (ca. 1.3 cm) above the main microscope stage and the Immobilizer bar should extend over the lens turret below.

When the InverterScope is attached to the lens turret, it is essential that the lens turret remain stable and not rotate. Immobilization of the lens turret is achieved via two strategies. First, as a preventative measure, the Zeiss ZEN software was configured to ignore objective lens selection on “Reuse.” This prevents automatic rotation of the lens turret when the user loads preset imaging configurations. Secondly, when the InverterScope is attached to the lens turret and is complete with a mounted objective lens, the weight of the InverterScope and objective lens could potentially cause position drift or turret drift/rotation by torqueing the lens turret. To rectify this a Rotation Immobilizer is used to stabilize the InverterScope on the turret and prevent rotation (**Figure 4A**). The Rotation Immobilizer physically prevents the InverterScope from moving the lens turret by blocking InverterScope rotation when attached. In our hands this is largely a preventative measure as attachment of the InverterScope does not add enough weight to rotate the lens turret on its own when using our chosen objective lens. It is acknowledged that using a heavier lens may affect the stability thus making the Rotation Immobilizer more functionally necessary. The Rotation Immobilizer can be purchased along with the InverterScope from LSM Tech. Affixing the Rotation Immobilizer is done before the InverterScope is attached to the lens turret.

**FIGURE 5:**
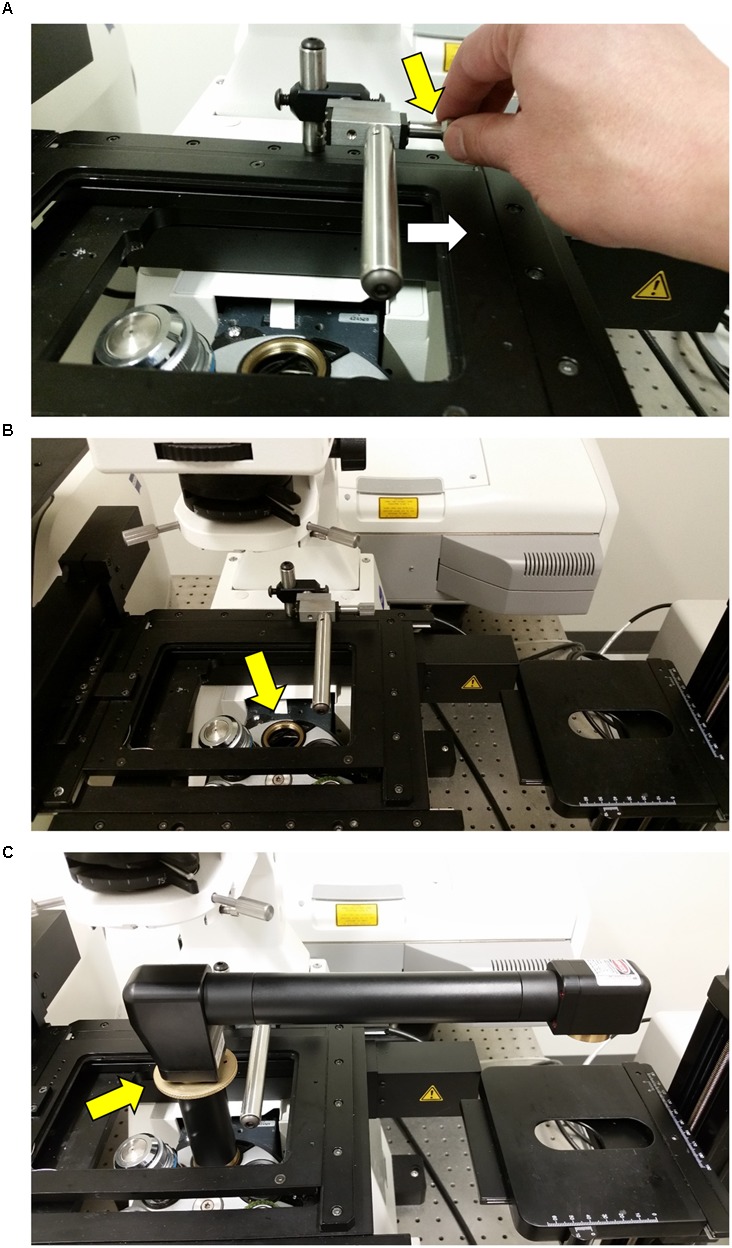
**Attaching the InverterScope to the lens turret. (A)** Before attaching the InverterScope to an empty port on the objective lens turret, the bar on the Rotation Immobilizer must be moved out of the way. This is accomplished by turning the Screw nut (yellow arrow) on the Rotation Immobilizer (**Figure 4**) until the Immobilizer bar is clear of the InverterScope attachment space (white arrow). **(B)** At this point the empty objective lens port should be not be obstructed. **(C)** The external threads on the base of the InverterScope (**Figure 1**) are then aligned with the objective lens port and the InverterScope is gently screwed into place by rotating the ring on the InverterScope shaft (yellow arrow). Do not over tighten. At this point the InverterScope will sit freely on the objective turret and the InverterScope arm will extend past the main microscope stage with the objective end of the InverterScope (**Figure 1**) posited directly over the XYZ Side Sample stage (**Figure 2**).

**FIGURE 6:**
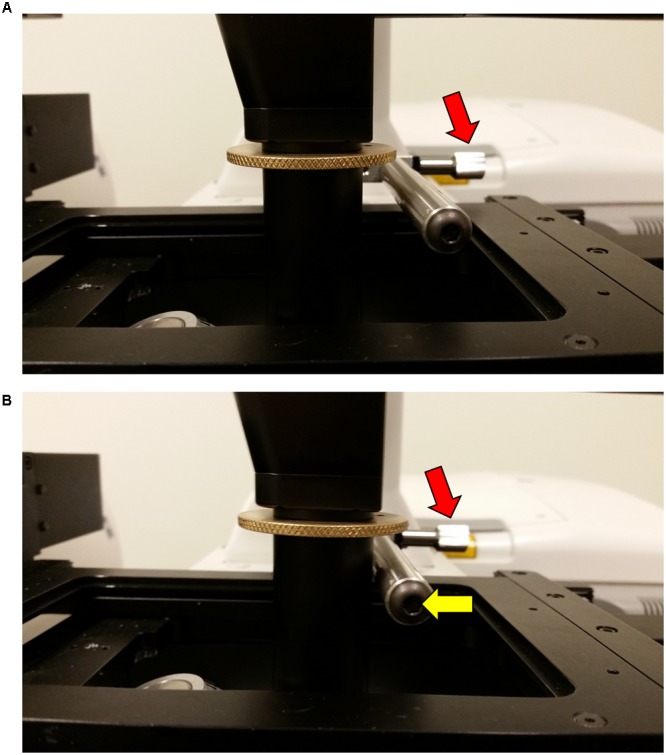
**Securing the InverterScope position with the Rotation Immobilizer bar.** To secure the InverterScope and prevent torque or drift, the bar on the Rotation Immobilizer is used to physically prevent InverterScope movement. In the “out” position **(A)**, the Immobilizer bar is not touching the shaft of the InverterScope. By turning the Immobilizer screw nut, the Immobilizer bar is positioned to just touch the InverterScope shaft **(B)**. It is important to not push the Immobilizer bar into the InverterScope shaft.

Before first use, a mounting stub for the Rotation Immobilizer was mounted on the rear of the main microscope stage (**Figure 4B**). This mounting stub is permeant, small, and does not interfere with standard imaging in any configuration. Once the mounting stub is attached to the microscope stage, the Rotation Immobilizer is simply slid onto the mounting stub and lightly tightened with an Allen key (**Figure 4C**). The Rotation Immobilizer must be positioned on the right of the mounting stub so that it aligns to the right of the InverterScope tube when the InverterScope extends to the right of the microscope stage (**Figure 4B**). The Rotation Immobilizer is mounted to height of approximately 0.5 inches above the main microscope stage. Once attached to the mounting stub, the Rotation Immobilizer bar can be positioned along the *X*-axis parallel to the main stage via rotating the screw nut on the Rotation Immobilizer (**Figures 5**, **6**). Before the InverterScope is attached to the lens turret the Rotation Immobilizer bar is positioned away from the lens turret by turning the screw nut (**Figures 5A,B**). This creates physical space to allow the InverterScope to be attached to the lens turret.

### Step 3: Affixing the InverterScope to the Lens Turret

The InverterScope is screwed into the lens turret as any objective lens would be using threads on the base of the InverterScope shaft. Below are the sequence of events for the attachment.

(1) Remove an objective lens from the position on the objective turret to be used for InverterScope and store safely (assuming it is not the objective lens to be used for imaging).
(2) Remove the InverterScope from the protective carrying case and remove by unscrewing the two protective caps on either end of the InverterScope.
(3) Placing the threaded base tube of the InverterScope into the open objective lens port (**Figure 1**), align the threads on the InverterScope with the lens port and then rotate the shaft of the InverterScope using the knurled dial on the InverterScope shaft until slight resistance indicates the InverterScope tube is fully engaged in the objective turret (**Figure 5C**). Do not over tighten, finger tight is sufficient. At this point the InverterScope should sit upright on the lens turret freely on its own.
(4) Secure the InverterScope with the Rotation Immobilizer by turning the Rotation Immobilizer screw nut until the Rotation Immobilizer bar just touches the InverterScope shaft (**Figure 6**). Do not “push” the Rotation Immobilizer bar into the shaft (**Figure 6B**). At this point the InverterScope is stabilized and will not rotate the lens turret. In nearly 100 h of imaging we have not had an incidence of the InverterScope moving or torquing the lens turret if attached in this manner.

### Step 4: Positioning of Samples of Side Stage and Imaging

**FIGURE 7:**
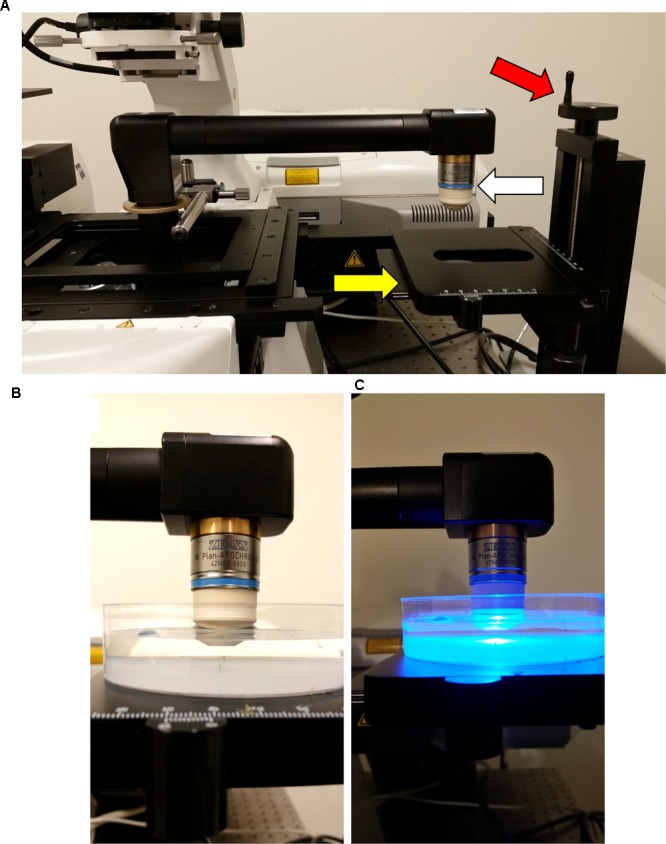
**Final configuration of the attached InverterScope. (A)** To complete the InverterScope attachment the desired objective lens (white arrow) is screwed into place on the objective end of the InverterScope. Samples are then placed under the objective lens on the sample stage (yellow arrow). With this model stage, the stage can be lowered or raised as necessary by rotating the stage control (red arrow). **(B,C)** Samples are then imaged in an aqueous environment using a water immersion “dipping” lens. Sample preparation is described in detail in (Prunet et al., 2016).

At this point the InverterScope arm should extend beyond the main microscope stage and be positioned above the XYZ Side Stage (**Figure 7A**). The final step prior to imaging is to attach the desired objective lens to the InverterScope (**Figure 7**). Imaging of living shoot meristems is done in an aqueous environment in most scenarios and for most of our imaging a 40× water immersion “dipping” lens is used (**Figures 7B,C**), but this is an individual preference (Prunet et al., 2016). Either detached or attached meristems can be imaged. Samples, plant growth, dissection, and mounting are performed as previously described (Prunet et al., 2016). In both cases, shoot meristems to be imaged are placed upright in dishes in an aqueous solution and the objective lens is lowered into the solution by moving the side stage up or down using the coarse stage height adjustment wheel (**Figure 7**). Care should be taken to not trap air bubbles on the objective lens (Prunet et al., 2016), and the objective lens may have to be lowered and raised as necessary to remove bubbles. Once the meristem is in approximate focal distance from the lens, further focusing is accomplished by using the microscope controls for standard objective lens focusing.

### Step 5: Disassembly and Removal of the InverterScope Attachment

One of the main advantages of using the InverterScope is the ease of detachment. Following imagining, the steps for attachment of the InverterScope are simply followed in reverse. Once imaging is complete, samples are removed and the Objective lens, InverterScope, and Rotation Immobilizer are removed in reverse order of their attachment and stored correctly back in their protective cases. The condenser is then moved back in position and reaffixed to the condenser mount.

### Considerations with Imaging Apical Meristems with the InverterScope

**FIGURE 8:**
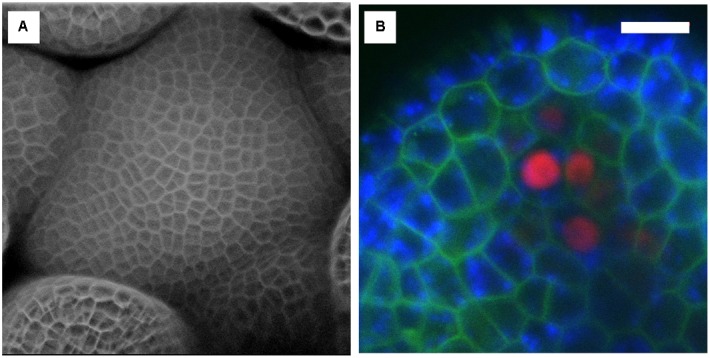
**Examples of confocal images obtained with the InverterScope.** **(A)** Surface reconstruction of Arabidopsis inflorescence meristem taken from a *Z*-series images using the InverterScope and reconstructed using ZEN software. Cell walls were stained with propidium iodide to visualize cell walls as described in (Nimchuk, 2017). A 40× water dipping objective lens was used with propidium iodide imaged at Ex514, Em 602-670. No segmentation software was used in image processing. **(B)** Three channel imaging of developing Arabidopsis sepal phloem cells (red nuclei, *pBAM3p::Ypet-N7*), chlorophyll (blue), and GFP tagged BAM1 receptor kinase (*pUBQ10::BAM1-2xGFP*) obtained by crossing plant line plant lines previously described in (Nimchuk et al., 2015). White bars, 10 μM. Imaging was performed with a 40× dipping objective lens with the following settings (GFP Ex488, Em 490-516; Chlorophyll Ex488, Ex602-729, Ypet, Ex514, Em519-548). For more examples of imaging obtained with the InverterScope see (Nimchuk, 2017).

One major consideration regarding the usage of the InverterScope is that inverting the light path from a standard inverted microscope results in an inversion of focus direction in the *Z*-plane. When compared to standard inverted imaging technique, focusing “down” becomes focusing “up” and vice versa. While this does not interfere with imaging acquisition or image quality, it does require the careful attention of a user who is more accustomed to imaging on the standard inverted configuration. In addition, use of the InverterScope does not affect the ability to acquire Z-stack image sets – user defined upper and lower Z-stack limits are designated in ZEN software as usual (**Figure 8**). The other consideration to bear in mind is that the positioning of the InverterScope hardware on the objective lens turret will render collection of transmitted light images impossible because the light is diverted from the normal transmitted path and thus does not reach the transmitted light detector. For our experience we have not had a need to collect visible light images of SAMs with our microscope as we are typically staining cells, monitoring fluorescent protein expression, or detecting chlorophyll. Typically we first focus on the SAM samples using a laser illumination that will not bleach our protein of interest, before performing actual imaging of interest.

## Discussion

**FIGURE 9:**
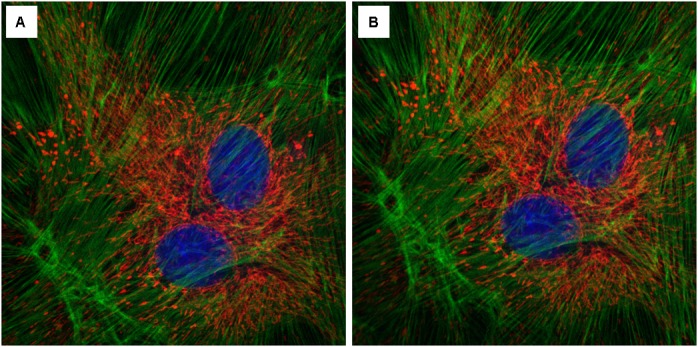
**Effect of the InverterScope on light intensity compared to standard inverted confocal imaging configuration. (A,B)** Three channel imaging of fixed Bovine Pulmonary Artery Epithelia Cells stained with DAPI (blue), Alex488-Phalloiding (green), and MitoTracker CMSRoS (red). **(A)** Image obtained on ZeissLSM710, **(B)** same sample, imaged with InverterScope attachment on ZeissLSM710. The same 63× Oil immersion objective lens was used for both images and the same imaging settings were used in both images (DAPI Ex405, Em 410-466; Alexa488 Ex488, Em 495-563; MitoTracker Ex560, Em 574-657).

Among the many technical challenges in imaging growing shoot meristems is the requirement of an upright imaging system, the lack of which prevents many plant labs from pursuing live imaging studies or forces them to adopt alternate imaging setups that are often not robust or introduce potentially confounding treatments. Here we demonstrate that an InverterScope attachment can be employed to modify more readily available inverted imaging systems to image growing apical plant tissues with ease and with minimal additional technical modifications. The InverterScope is fully detachable and can be used on any inverted microscope system. Purchasing an upright confocal imaging system can typically costing in the range of $500,000 or more, well out of the resource range for many labs or departments. In contrast the InverterScope and Rotational Immobilizer cost just over $7,000, well within the range of most lab budgets. In theory, use of the InverterScope should result in some loss of light, owing to additional path length and use of two additional reflective mirrors in the InverterScope joints. However, in our experience this has not prevented the imaging of any of our lines and images are easily obtained for most lines (**Figure 8**), and see (Nimchuk, 2017). In addition we have not noticed a significant effect on light intensity in experiments comparing standard inverter imaging and our InverterScope using fixed tissue slides (**Figure 9**). In five separate imaging attempts, images obtained with the InverterScope were brighter in two, weaker in two, and comparable in one to the sample samples imaged with our standard inverted confocal. Our InverterScope was used in a recent publication on meristem function demonstrating the robustness of the method (Nimchuk, 2017). One of the significant advantages of the InverterScope is that the successful images of SAMs can be obtained at a close to 100% rate. In contrast, tissue damage, sample desiccation and focal plane issues that arise with imaging without the InvertScope on our ZeissLSM710 make obtaining images possible about 10–20% of the time. This significant improvement in success rate with the InverterScope allow large scale imaging experiments to proceed in a timely and cost effective manner.

While the 400S model InverterScope rotates the light path by 180°, each joint is flexible and can be rotated allowing the InverterScope to be rotated 90°. As such the InverterScope can also be used to image upright growing plants from the side. All that would be required to accomplish this goal would be to have plants mounted on the side stage at the appropriate distance and orientation. The stage of the side sample stage (**Figure 2**, XYZ Side Stage, LSM Tech) also allows for rotation of the stage for easy side imaging. As such, the Inverted allows not only upright imaging of meristems, but also sideways imaging of vertically growing plants. We recognize that there are many different microscope configurations and brands but the adaptability and modularity of most microscope configurations should allow use of an InverterScope.

## Author Contributions

ZN and TP conceived the experiments. TP ordered and attached the accessory components. ZN performed all imaging and InverterScope attachment. ZN and TP wrote the manuscript.

## Conflict of Interest Statement

The authors declare that the research was conducted in the absence of any commercial or financial relationships that could be construed as a potential conflict of interest.

## Footnotes

1 http://lsmtech.com/
